# Spray-Drying Microencapsulation of Pink Guava (*Psidium guajava*) Carotenoids Using Mucilage from *Opuntia ficus-indica* Cladodes and Aloe Vera Leaves as Encapsulating Materials

**DOI:** 10.3390/polym14020310

**Published:** 2022-01-13

**Authors:** María Carolina Otálora, Andrea Wilches-Torres, Jovanny A. Gómez Castaño

**Affiliations:** 1 Grupo de Investigación en Ciencias Básicas (NÚCLEO), Facultad de Ciencias e Ingeniería, Universidad de Boyacá, Tunja 050030, Boyacá, Colombia; andreawilches@uniboyaca.edu.co; 2 Grupo Química-Física Molecular y Modelamiento Computacional (QUIMOL®), Facultad de Ciencias, Universidad Pedagógica y Tecnológica de Colombia (UPTC), Avenida Central del Norte, Tunja 050030, Boyacá, Colombia

**Keywords:** guava, carotenoids, microencapsulation, mucilage, spray-drying, aloe vera, *Opuntia ficus-indica*

## Abstract

In this work, the capacity of the mucilage extracted from the cladodes of *Opuntia ficus-indica* (OFI) and aloe vera (AV) leaves as wall material in the microencapsulation of pink guava carotenoids using spray-drying was studied. The stability of the encapsulated carotenoids was quantified using UV–vis and HPLC/MS techniques. Likewise, the antioxidant activity (TEAC), color (CIEL*ab*), structural (FTIR) and microstructural (SEM and particle size) properties, as well as the total dietary content, of both types of mucilage microcapsules were determined. Our results show that the use of AV mucilage, compared to OFI mucilage, increased both the retention of β-carotene and the antioxidant capacity of the carotenoid microcapsules by around 14%, as well as the total carotenoid content (TCC) by around 26%, and also favors the formation of spherical-type particles (Ø ≅ 26 µm) without the apparent damage of a more uniform size and with an attractive red-yellow hue. This type of microcapsules is proposed as a convenient alternative means to incorporate guava carotenoids, a natural colorant with a high antioxidant capacity, and dietary fiber content in the manufacture of functional products, which is a topic of interest for the food, pharmaceutical, and cosmetic industries.

## 1. Introduction

Guava (*Psidium guajava* L.) is a tropical fruit native to Central America, northern South America, and the Caribbean that has spread to many other tropical and subtropical regions, including southern North America, southwestern Europe, tropical Africa, Oceania, and south and southeast Asia. Its annual production is estimated at more than 55 million tons worldwide, with India being the largest producer at around 45% of the total production [1].

Guava is a rich source of vitamin C and dietary fiber (hemicellulose, pectin, cellulose, and lignin), and has a lower content of other micronutrients such as vitamins A, B1 (thiamine), B2 (riboflavin), B3 (niacin), and B5 (pantothenic acid). In addition, it is a good source of phosphorus, calcium, iron, potassium, and sodium [2,3]. A single guava fruit can provide as much as 250% of the required daily value of vitamin C [3]. Furthermore, this fruit has been recognized as a potential source of functional compounds with high antioxidant capacities, such as tannins, phenols, triterpenes, flavonoids, and carotenoids [4,5].

Pink guava, a pink-fleshed fruit known as the “apple of the tropics” due to its attractive color, aroma, and flavor characteristics, has been considered an ideal source of β-carotene, γ-carotene, β-cryptoxanthin, rubixanthin, lutein, cryptoflavin, phytofluene neochrome, and lycopene [6,7]. Among these, lycopene (C_40_H_56_) is the aliphatic hydrocarbon-type carotenoid responsible for the reddish hue of the pink guava pulp. This compound is also attributed to the beneficial effects associated with human health related to the prevention of cardiovascular and degenerative diseases, cancer, diabetes, and inflammation, among others [8,9,10]. However, lycopene is prone to undergoing isomerization and oxidation reactions when handled in isolation in the presence of oxidants (air and water), light, and temperature, which leads to a significant loss of its bioactive and physicochemical properties [11]. These factors, in addition to its poor solubility in aqueous media and ensuing low bioavailability, restrict its application as a functional bio-ingredient [12].

Microencapsulation using biopolymers as structural materials has been used in the food, pharmaceutical, and cosmetic industries. This process is both an economic and natural strategy used to safely contain and deliver sensitive active or volatile compounds while isolating them from external factors such as temperature, oxygen, light, humidity, pH variations, macromolecules, and metabolites. This ensures their bioavailability and antioxidant capacity; however, to ensure the effectiveness and stability of the encapsulation, a critical aspect that must be properly designed is the structuring of the biopolymeric wall [13,14].

Natural hydrocolloids such as mucilage, gums, and gelatins have been shown to be functional biopolymers that possess an intrinsic antioxidant capacity and high dietary fiber content. These compounds also contain suitable physicochemical properties for use as encapsulating wall materials [15,16,17,18,19]. The mucilage extracted from the cladodes of *Opuntia ficus-indica* (OFI) is a heteropolysaccharide with thickening, binding, emulsifying, stabilizing, and gelling properties. It is rich in dietary fiber (73.9 g/100 g of powdered mucilage), which has been used as a wall material in different encapsulation formulations [20,21]. OFI mucilage has a molar mass of 3 × 10^6^ gmol^−1^ and is composed of L-arabinose (24.6–42.0%), D-galactose (21.0–40.1%), D-xylose (22.0–22.2%), L-rhamnose (7.0–13.1%), and α-D-(1 → 4) galacturonic acid (8.0–12.7%) [16]. Likewise, the mucilage extracted from the hydroparenchyma of aloe vera (AV) leaves is a polysaccharide that is considered an excellent wall material to be applied in the encapsulation of active ingredients due to its thickening and emulsifying properties [15,16]. This biopolymer is rich in dietary fiber (37.0 g/100 g of powdered mucilage) and is mainly composed of acemannan, a polysaccharide with a molecular weight of around 40–50 kDa. It contains large amounts of partially acetylated mannose units (>60%), glucose (∼20%), and galactose (<10%) [16].

To date, there are few studies focused on the effect of encapsulation on the bioavailability and conservation of the antioxidant capacity of the carotenoids extracted from the guava fruit, using different wall biomaterials. Leite et al. recently demonstrated that lipid core nanocapsules, using polysorbate 80 coated poly-ɛ-caprolactone as the wall material, could be efficiently applied to stabilize the lycopene-rich extract of red guava. The lipid core nanocapsules optimized the stability of lycopene at 5 °C for 7 months, improved its toxicity against MCF-7 cancer cells, inhibited the production of intracellular ROS and NF-κB in human microglial cells, and did not impact the integrity of the erythrocyte membrane [13]. Chaves et al. more recently prepared microcapsules loaded with guava pulp by means of the spray-drying (SD) technique, using a mixture of inulin and maltodextrin as functional encapsulating material [22]. They found that microcapsule formulation with a higher percentage of inulin resulted in a better retention and stability of the antioxidant activity of the pulp over time. It also resulted in a higher retention of the carotenoid content and a more stable microstructure. Previously, Osorio et al. encapsulated the aqueous extract of pink-fleshed guava fruit by SD using maltodextrin, arabic gum, and their mixtures as wall materials [23]. SEM observations verified the production of spherical microencapsulates, while thermal analyses revealed a higher thermal stability of the maltodextrin particles.

In this study, we investigated the influence on the physicochemical and antioxidant properties of pink guava carotenoids given by the mucilage of OFI cladodes and AV leaves when used as the wall material in microencapsulation processes using the SD technique. The structural (FTIR), microstructural (SEM and particle size), and thermal (DSC/TGA) properties, as well as the total dietary fiber content, were determined for each type of microcapsule formulation, i.e., SD-OFI and SD-AV. For this study, Colombian pink guavas were used, whose current production is estimated at around 550,000 tons per year. This makes Colombia rank 17th among the world producers of this fruit [1].

## 2. Materials and Methods

### 2.1. Reagents

Acetonitrile and methanol solvents (HPLC grade), as well as acetone and ethanol solvents (analytical grade), were purchased from Merck (Darmstadt, Germany). 2,2′azino-bis (3-ethylbenzthiazoline-6-sulfonic acid) (ABTS) was purchased from Sigma Aldrich (St. Louis, MO, USA).

### 2.2. Vegetal Materials

Guava fresh fruits (*Psidium guajava* L.) of the Palmira ICA-1 regional variety were purchased at a local supermarket in the city of Tunja in the department of Boyacá, Colombia. The fruits were selected based on their uniform size, firmness, color, and absence of defects (spots, depressions, or cracks). The fruits were washed with distilled water and manually peeled to obtain the pulp (edible part) and seeds. The edible portion was immediately chopped and homogenized in a blender at minimum power for 1 min. Then, the pulp was sieved to remove the seeds, maintained in an amber bottle to protect against photodegradation, and stored at 4 °C until its analysis. The guava pulp presented a pH of 3.85 ± 0.02 and soluble solids content of 8.00 ± 0.03 °Brix, determined with a digital pH meter (ORION™ Versa Star™, Thermo Scientific Inc., Waltham, MA, USA) and a digital hand refractometer (Boeco model 32395, Hamburg, Germany), respectively.

### 2.3. Characterization of Vegetal Material

The guava pulp was frozen at −80 °C in an ultra-low temperature freezer (Buzzer, model MDF–86V188E, Shanghai, China) for 48 h. Then, the samples were freeze-dried in a Freezone 4.5 L freeze dryer (Labconco, Kansas City, MO, USA) at −84 °C in a vacuum of 0.13 mbar for 48 h. After freeze-drying, the samples were triturated using a food processor and stored in amber bottles until further analysis. The non-microencapsulated lyophilized pulp of guava (LGP) was designated as a control sample.

### 2.4. Extraction of Wall Materials

For the extraction of mucilage from cladodes of *Opuntia ficus-indica* and aloe vera leaves, the methodology reported by Quinzio et al. [24] and Otálora et al. [16] was used.

The cladodes and aloe vera leaves were washed with distilled water at room temperature, after which the epidermis of each plant material was carefully separated from the inner pulp (i.e., the medulla) using a Teflon knife.

The medulla obtained from the cladodes was cut into small pieces and placed in a 1000 mL beaker. Distilled water at room temperature was added to the beaker in a 1:2 *v*/*v* (medulla:water) ratio. The mixture was left for 24 h and manually squeezed to extract the gel. Similarly, the medulla obtained from aloe vera leaves was also cut into small pieces and manually squeezed to extract the gel.

Each of the gels obtained from the cladodes and aloe vera leaves were filtered through a nylon cloth, separately. Then, 95% ethanol was added to each of the filtered gels in a ratio of 3:1 (ethanol:centrifuged gel) at room temperature. The mixture was then stirred manually with a glass rod until the appearance of a white-milky gel (precipitated mucilage) was present.

The mucilage gels obtained from the cladodes and aloe vera leaves were placed in Petri dishes and dried in an oven (UM 400, Memmert, Schwabach, Germany) for 24 h at 60 and 105 °C, respectively. The dried materials were manually macerated in a porcelain mortar until a fine powder was obtained. The OFI (obtained from cladodes) and AV (obtained from aloe vera leaves) powdered mucilages were placed in separate high-density polyethylene bags and stored in a desiccator at room temperature with a relative humidity of 30% until use.

### 2.5. Preparation of Microcapsules 

A mass of 1.2 g of mucilage obtained from Opuntia ficus-indica cladodes and 0.4 g of mucilage obtained from aloe vera leaves were each dissolved separately in 100 mL of distilled water at 18 °C. To ensure complete solubilization, both solutions were constantly stirred at 300 rpm for 2 h using a magnetic stirrer (C-MAG HS 7 S000, IKA, Staufen im Breisgau, Germany). The lyophilized guava pulp was mixed separately with each mucilage solution in a ratio of 1:10 *w/v* (10 g:100 mL), respectively. In each case, the feed mixture was kept under constant magnetic stirring at room temperature until homogeneity was achieved. The total solids content of the feed mixes was 5.55% for SD-AV and 4.34% for SD-OFI. Each mixture was then fed into a mini spray dryer (B-290, Büchi Labortechnik, Switzerland) with aspiration maintained at 100% (35 m^3^/h) to maximize the separation rate of the cyclone [25] and a compressed air pressure of 40 bar. The spray drier used a nozzle with an internal diameter of 0.7 mm, a feed flow of 350 mL/h, and an inlet air temperature of 120 °C. The two microencapsulates that were obtained from this procedure, SD-OFI (pulp/mucilage obtained from cladodes *Opuntia ficus-indica*) and SD-AV (pulp/mucilage obtained from aloe vera leaves), were stored in the dark at −20 °C for subsequent analysis and use.

### 2.6. Characterization of the Guava Pulp Microcapsules

#### 2.6.1. Carotenoids Quantification

The lyophilized guava pulp (LGP) and microcapsules (SD-OFI and SD-AV) were accurately weighed and dispersed in acetone, stirred for 1 min at room temperature, and filtered with a Millipore membrane (0.45 µm). UV-Vis analysis was run at 450 nm in order to quantify the β-carotene equivalent present in each of the samples (LGP, SD-OFI, and SD-AV) using a UV–vis spectrophotometer (V530, Jasco, Hachioji, Tokyo, Japan). The total carotenoid content was determined from the standard curve of β-carotene. The retention of total carotenoid in the microencapsulated was expressed as µg β-carotene/g of sample. 

#### 2.6.2. Lycopene and β-Carotene Analysis by HPLC–MS

The HPLC–MS analyses of the lyophilized pulp of guava (LGP), SD-OFI, and SD-AV microencapsulates were performed using an Acquity ultraperformance liquid chromatography (UPLC) system equipped with a Xevo TQD Mass Spectrometer (Waters Corp., Milford, MA, USA) equipped with an electrospray ionization (ESI) probe that was operated in positive ion mode. An Acquity UPLC^®^ T3 HSS C18 analytical column (2.1 mm × 100 mm, 1.7 µm particle size) was used for the analysis of analytes present in each sample with the column temperature being maintained at 40 °C and using a flow rate of 0.4 mL min^–1^. The solvent mixture was composed of acetonitrile (solvent A) and methanol (solvent B). The gradient elution program was set as follows: 0–5 min, 100% A; 5–8.5 min, 80% B and 20% A; and 8.5–12 min, 100% A. The injection volume was set at 2 μL. The MS parameters were as follows: capillary voltage was set at 2.5 kV while block and desolvation temperatures were set at 150 °C and 400 °C, respectively. Desolvation gas flow rate was set to 800 L h^−1^ and cone gas was set at 50 L h^−1^. Cone voltages were set to 68 and 64 V and collision energies were set to 60 and 52 eV for lycopene and β-carotene, respectively.

#### 2.6.3. Trolox Equivalent Antioxidant Capacity (TEAC)

Trolox equivalent antioxidant capacity (TEAC) was measured using the method reported by Re et al. [26]. The lyophilized guava pulp (LGP) and microcapsules (SD-OFI and SD-AV) were accurately weighed and dispersed in methanol, stirred at room temperature, and filtered with a Millipore membrane. The liquid samples were mixed with an ABTS^•+^ solution and its absorbance was read at 734 nm using a UV–vis spectrophotometer (V530, Jasco, Hachioji, Tokyo, Japan). Results were expressed in mmol Trolox equivalents/kg of sample (TEAC).

#### 2.6.4. Color Parameters

The CIEL*ab* parameters (L*, a*, b*) of lyophilized guava pulp (LGP) and microencapsulates SD-OFI and SD-AV samples were measured using a colorimeter (CM-5, Konica Minolta Sensing, Inc., Osaka, Japan). The other color parameters, chroma (C*ab) and hue (*hab*), were calculated using Equations (1) and (2).

C*_ab_ = [(a*)^2^ + (b*)^2^]^1/2^ (1)

*h_ab_* = arctan [b*/a*] (2)

### 2.7. Fourier-Transform Infrared (FTIR) Spectroscopy

The infrared spectra of the powdered mucilages of *Opuntia ficus-indica* (OFI) and aloe vera (AV) as well as microencapsulates SD-OFI and SD-AV were recorded on a Shimadzu Prestigie 21 spectrophotometer (Duisburg, Germany) equipped with a Michelson-type interferometer, a KBr/Ge beam-splitter, a ceramic lamp, and a DLATGS detector. The FTIR spectra were measured in the range of 4500–500 cm^−1^ with a resolution of 3.0 cm^−1^ and 30 cumulative scans using the attenuated total reflectance/reflection (ATR) technique.

### 2.8. Microstructural Characterization

#### 2.8.1. Scanning Electron Microscopy (SEM)

The microscopic morphology of the powdered mucilages (OFI and AV) as well as the microencapsulates (SD-OFI and SD-AV) were evaluated by scanning electron microscopy (SEM) using EVO MA 10-Carl Zeiss equipment (Oberkochen, Germany) operating at 20 kV. All samples were coated by gold–palladium sputtering before their examination.

#### 2.8.2. Particle Size

The size distributions and average diameters of the SD-OFI and SD-AV microencapsulates were determined using a laser diffraction particle size analyzer Mastersizer 3000 system equipped with a Hydro MV dispersion unit (Malvern Panalytical Ltd., Malvern, UK). A particle refractive index of 1.52, dispersant refractive index of 1.33, and an obscuration range of 5.18–5.32% were applied. Volume weighted mean diameter D[4,3] and area-volume mean diameter D[3,2] were obtained. The span of the volume-based distribution using droplet size was calculated considering an average diameter equivalent to 90%, 50%, and 10% of the cumulative volume. 

### 2.9. Thermal Characterization

Thermogravimetric analysis (TGA)/differential scanning calorimetry (DSC) of the *Opuntia ficus-indica* and aloe vera powdered mucilages, as well as microencapsulates, SD-OFI, and SD-AV were performed on a TA Instrument (SDT Q600 V20.9 Build 20, New Castle, DE, USA). Argon was used as a purge gas (100 mL/min). The dried samples of OFI and AV mucilages were placed in aluminum pans and heated from 20 to 600 °C at a heating rate of 10 °C/min.

### 2.10. Dietary Fiber Content

Total dietary fiber (TDF) contents in SD-FI and SD-AV microencapsulated samples were determined using a total dietary fiber test kit (TDF-100A), provided by Sigma Aldrich (St. Louis, MO, USA), which is based on the enzymatic–gravimetric method AOAC 985.29 [27].

### 2.11. Statistical Analysis

Physicochemical data, as presented in Table 1, were reported as the mean ± standard deviation (*n* = 3). The Kruskal–Wallis test was performed to identify differences among the means using InfoStat/P version 1.1 statistical software. Differences at probability level *p* < 0.05 were considered significant.

## 3. Results and Discussion

### 3.1. Total Carotenoid Content, Antioxidant Capacity, Dietary Fiber Content, and Color Parameters

Table 1 shows the physicochemical parameters of total carotenoid content (TCC), antioxidant capacity (TEAC), dietary fiber content, and color (CIEL*ab*) of the pink guava pulp microcapsules (SD-OFI and SD-AV), together with the respective values for the lyophilized unencapsulated sample (LGP).

As shown in Table 1, spray-drying microencapsulation of the guava pulp using AV and OFI mucilage as the wall material led to a decrease in the total carotenoid contents of around 78% and 84%, respectively, when compared to the unencapsulated (LGP) sample. This poor carotenoid retention after atomization can be attributed to the high temperature to which these thermolabile compounds were exposed during the spray-drying microencapsulation. These results agree with recent studies reported in the review by Santos [28]. Likewise, the stability of microencapsulated carotenoids may have been influenced by the temperature at which they were stored until their later physicochemical characterization. The final load of total carotenoids in the aloe mucilage microcapsule (SD-AV) resulted in a slightly higher amount (3.6%) compared to the cactus mucilage microcapsule (SD-OFI). This difference was correlated with the amount of total dissolved solids in the feed mixes (5.55% for SD-AV and 4.34% for SD-OFI) introduced into the spray-drying equipment. The total solids content impacts the viscosity of the feed mix, which influences the size of the atomized droplets and particles, and thus modifies the retention rate of carotenoids [28]. The slight increase in the total solid concentration in the SD-AV feed mixture led to an increase in its viscosity, which reduced the circulation of the pulp core within the droplets. It also reduced the formation time of the semi-permeable membrane around the core, resulting in a decrease in the loss of carotenoids by a migration towards the surface of the microparticle. Another phenomenon that could have contributed to the higher presence of carotenoids in the SD-AV microcapsule is the greater degree of interaction of the pulp biomolecules with the macromolecules of the AV mucilage [29].

Despite the significant total loss of carotenoids that occurred during the spray-drying of the guava pulp microencapsulation, the antioxidant capacity (TEAC) measured in SD-AV and OFI-AV microcapsules presented losses of only around 17% and 28%, respectively, compared to the LGP sample (Table 1). This result indicates that the main active principles responsible for the antioxidant capacities (of both the mucilage biopolymer and the guava pulp) were largely preserved during the spray-drying microencapsulation process. Since the amount of guava pulp in both feed mixes was the same, the significantly (*p* < 0.05) higher TEAC value in the SD-AV microcapsules compared to the SD-OFI microcapsules (26.8 vs. 23.2 mmol equivalents of Trolox/kg of sample on a dry basis, respectively) can be attributed to the cooperative effect resulting from the lycopene content of pink guava and phenolic content of AV mucilage [30] (Section 3.3). Similar core-wall contributing effects on antioxidant capacities have been observed in microcapsules of betaxanthin from *Opuntia megacantha* fruits using a mixture of maltodextrin and cactus cladode mucilage as the encapsulating agent [31].

The color parameters listed in Table 1 show how the luminosity value (L*) (i.e., the light/bright appearance) for the SD-OFI microcapsules was higher than the values obtained in both the lyophilized guava pulp and the aloe vera microcapsule. This is possibly due to the whiter color coming from the higher ratio in weight of OFI mucilage in relation to the guava pulp (pulp:mucilage ratio of 1:0.12 for SD-OFI vs. 1:0.04 for SD-AV) used in the feed mixture for this microencapsulation. The range of values of the a* and b* parameters indicate that the color of both the microcapsules and the lyophilized guava pulp was framed in red-yellow hues. The chroma parameter (Cab*) was significantly lower (*p* < 0.05) for the SD-OFI microcapsules (i.e., lower a* and b* values) when compared to the SD-AV microcapsules. This behavior could be correlated with a higher degradation rate of lycopene (pink pigment), which would coincide with the lower antioxidant capacity of the SD-OFI microcapsule. As shown below, this greater loss of lycopene in the SD-OFI microcapsules was confirmed by HPLC-MS quantification (Section 3.2) and could be correlated with their more cracked microscopic morphology (Section 3.4). Finally, the parameter (h_ab_*) agreed with a yellowish hue for both types of microcapsules (77.19 for SD-OFI and 77.27 for SD-AV). This was associated with the average size determined for these particles (25.9 µm for SD-OFI and 26.4 µm for SD-AV, see Section 3.4). This result coincides with the reported effect of increasing the value of h_ab_* as a function of a decrease in the particle size [31].

As reported by other studies [22,23], the nature and concentrations of the wall materials are variables that play an important role in the color parameters of microcapsules produced by spray-drying. Shishir et al. observed a decrease in the a*/b* value for microcapsules produced with DE 10 maltodextrins as the encapsulating material [22]. Meanwhile, Osorio et al. reported an increase in color parameters (+a*, +b*) in the production of microcapsules using DE 19–20 maltodextrin as the encapsulating agent [23]. In the present work, the concentration of OFI and AV mucilages did influence the chromatic parameters (Cab*) of the microcapsules. However, the difference in the color parameters between the microecapsulation studies using maltodextrin and those carried out in the present work may have been related to the fact that maltodextrin is a dextrinization of starch, in that it influences the dextrose equivalent (DE), while that mucilage is a polysaccharide.

The total dietary fiber content of 9.3 g/100 g of sample was greater in the SD-OFI microcapsules when compared to the SD-AV microcapsules. This behavior could be associated with the concentration of the mucilage, and therefore, the total dietary fiber content in the feed-mixture, since the amount of guava pulp was the same. Camacho, et al. [32] reported a total dietary fiber content in pink guava pulp, Palmira ICA-1 regional variety, of 5.42 ± 0.07%. Otálora et al. [16] determined a total dietary fiber content *Opuntia ficus indica* and aloe vera powder mucilage of 73.9 g/100 g of sample and 37.0 g/100 g of sample, respectively. Therefore, both types of microcapsules could be incorporated into food products with health benefits for consumers.

### 3.2. HPLC-MS Identification Analysis

**Figure 1:**
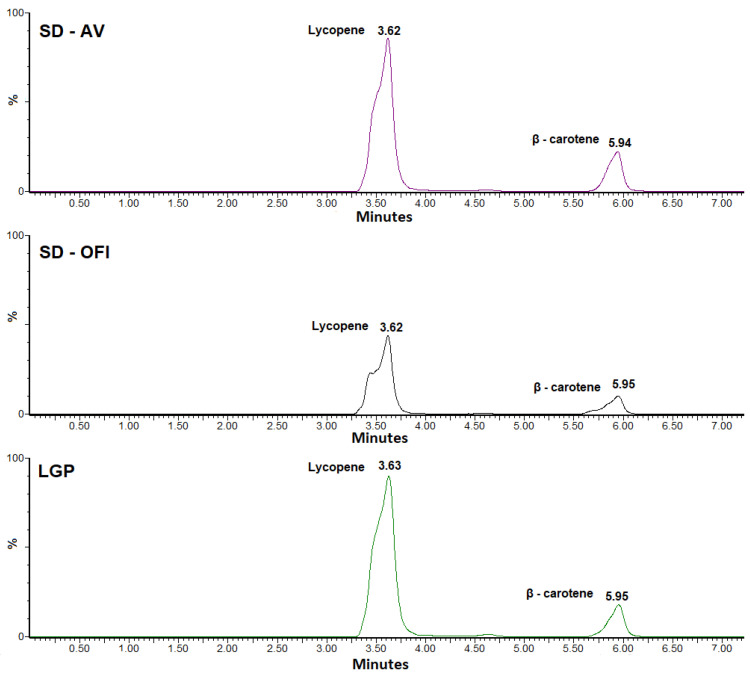
HPLC-MS chromatograms of SD-OFI and SD-AV guava pulp microcapsules and lyophilized guava pulp (LGP).

The determination of the contents of lycopene and β-carotene in the SD-OFI and SD-AV guava pulp microcapsules and the lyophilized guava pulp (LGP) sample was carried out using HPLC-MS, the chromatograms of which are shown in Figure 1.

The quantification of β-carotene by HPLC-MS shows a minimal variation in its content between the three types of samples analyzed (179 for LGP, 181 for SD-OFI, and 211 mg/kg dry sample for SD-AV). This suggests a small effect on this component during the spray-drying process. The slightly lower β-carotene content in the LGP sample compared to the SD-AV and SD-OFI microcapsules may have be related to the protective effect against oxidative processes provided by the mucilage biomaterial. The microscopic structure of LGP is characterized by an irregular and angular morphology (Section 3.4) that could contribute to the diffusion of oxygen (β-carotene degradation factor) in contrast to the structure of the mucilage microparticles that provide a barrier effect against oxidation of the carotenoids loaded in their interior [29,33]. The SD-OFI microcapsules showed a lower level of β-carotene retention compared to the SD-AV microcapsules which was attributed to the greater internal porosity and lower wall thickness of the OFI mucilage wall (*vide infra*). This structural difference was what allowed for a greater degree of oxygen diffusion. The more irregular microstructure of the SD-OFI encapsulating matrix may be have been related to the higher viscosity of the emulsion entering the spray-drying equipment (see Section 3.1). This can cause a longer time for the formation of the OFI mucilage film around the droplets as well as a greater exposure to heat during the drying process [34,35]. Similar behavior was observed in lemon essential oil microcapsules using a mixture of mesquite gum and chia mucilage as a wall material [36].

In contrast, significantly lower amounts of lycopene were found in the SD-OFI (4155 mg/kg dry sample) and SD-AV (4104 mg/kg dry sample) microcapsules compared to the amount determined in the lyophilized pulp sample (9281 mg/kg). This showed a loss of approximately 55% of the lycopene content by thermal effects (Section 3.1) during the guava pulp microencapsulation process by spray-drying in both cases.

### 3.3. Fourier Transform Infrared Spectroscopy (FTIR)

**Figure 2:**
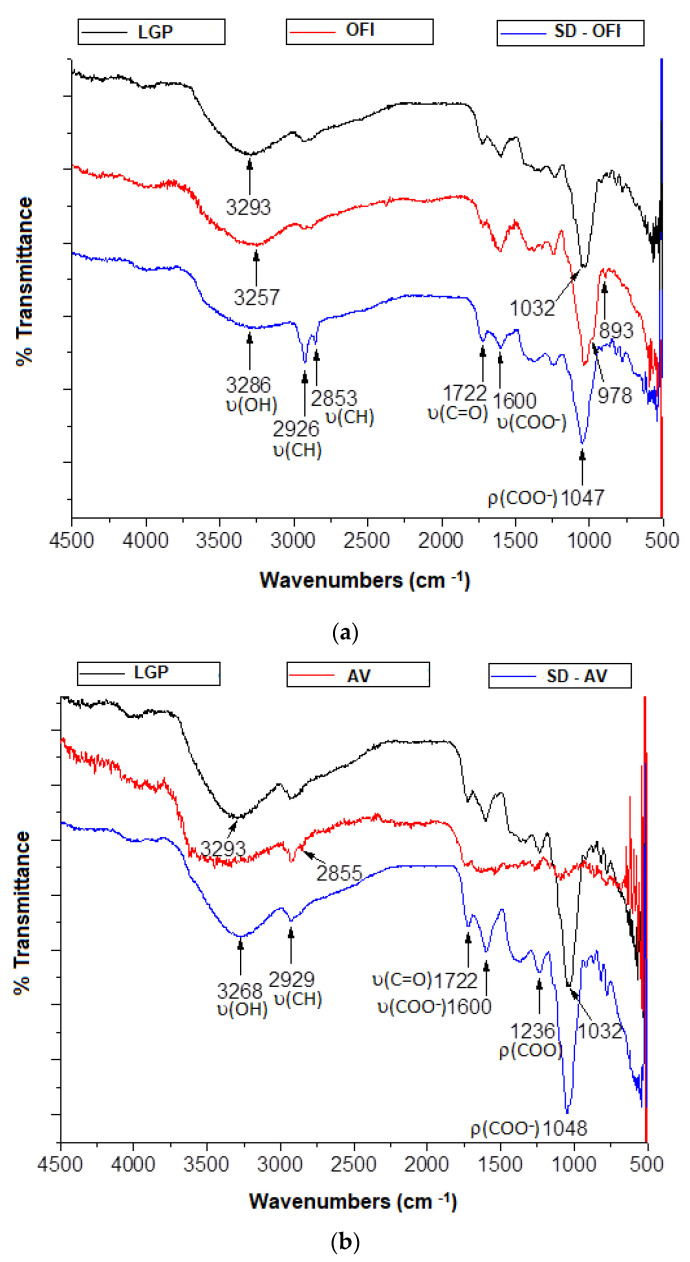
FTIR spectra of lyophilized guava pulp (LGP), mucilages ((**a**) OFI and (**b**) AV) and microcapsules (SD-OFI and SD-AV).

The FTIR spectra of lyophilized guava pulp, SD-OFI and SD-AV microcapsules, as well as the respective spectra of the free OFI and AV mucilage, are presented in Figure 2a,b.

The main infrared absorbances observed in the microcapsules are attributable to the most representative functional groups present both in the OFI or AV mucilage as well as in the guava pulp. The band at 3268 and 3286 cm^−1^ observed in the FTIR spectra of the SD-AV and SD-OFI microcapsules, respectively, was attributed to a combined contribution of the hydroxyl group from both R-OH and the C(O)-OH moieties involved in intramolecular hydrogen bonds. The absorptions observed at 2929 (SD-AV) and at 2926 and 2853 cm^−1^ (SD-OFI) were assigned to the aliphatic C–H stretch. Meanwhile, the signals at 1722 and 1600 cm^−1^, observed in both microcapsule FTIR spectra, were attributed to the carbonyl (C=O) and COO- stretching modes, respectively, of the *D*-galactopyranosyluronic acid residues. The position of the carbonyl signal at 1722 cm^−1^ in both microcapsule spectra was associated with the interaction of the carbonyl of the acetyl residue in mucilage and the C=O stretching groups in the hemicellulose, pectin, and lignin structures in the pulp of guava. A similar behavior was observed in gallic acid microcap19sules using aloe vera as a wall material [15]. On the other hand, the intense signals at 1047 (SD-OFI) and 1048 cm^−1^ (SD-AV) were related to the compound’s movement between the polysaccharide skeleton and the stretching vibration of the C–O flexion, indicating the presence of alcohols, ethers, esters, and carboxylic acids which are mainly linked to phenolic acids and flavonoids [16,37]. These spectroscopic results suggest that guava molecular structures and wall materials are preserved during the microencapsulation process and that no new chemical bonds were formed. In other words, the carotenoids present in the guava pulp were physically trapped in the AV and OFI mucilage matrix, and only hydrogen bonds and van der Waals interactions were formed [38].

### 3.4. Microscopy Morphology and Particle Size

**Figure 3:**
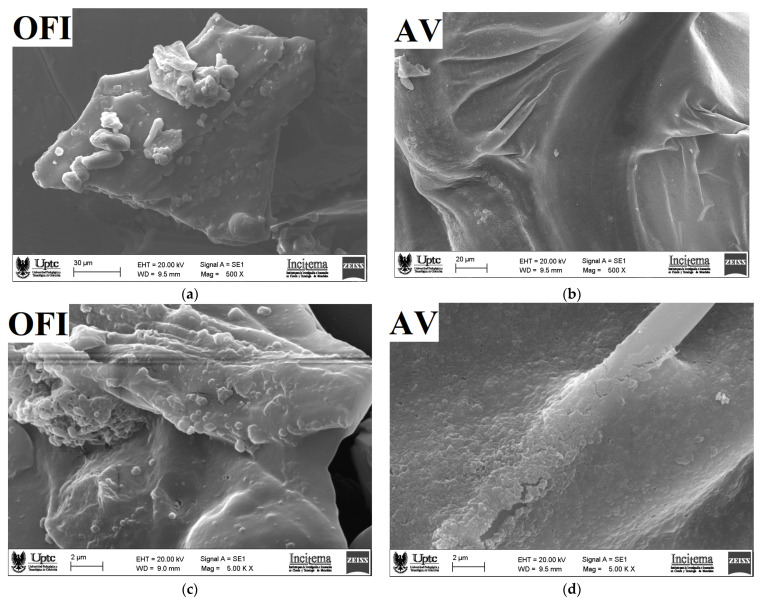
SEM micrograph images of the surface at 500× (**a**,**b**) and 5000× (**c**,**d**) of mucilages of OFI and AV in powder, respectively.

**Figure 4:**
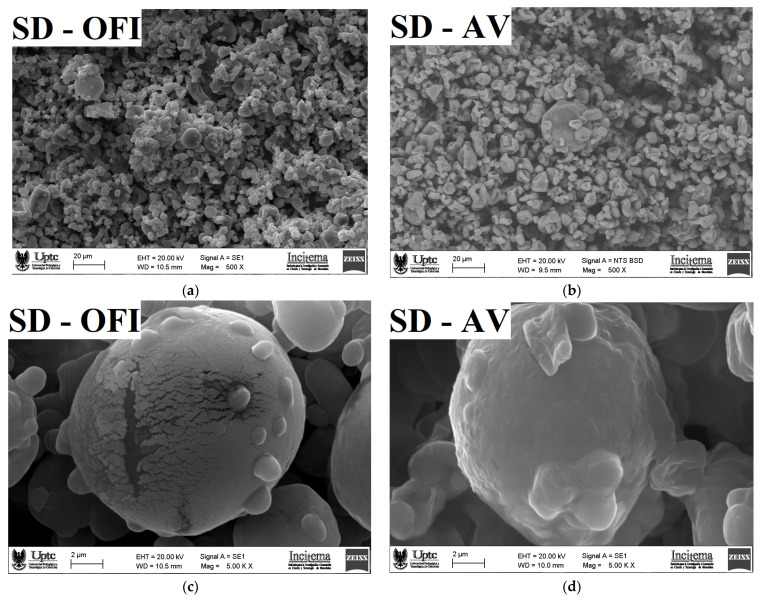
SEM micrograph images of the surface at 500× (**a**,**b**) and at 5000× (**c**,**d**) of SD-SD–OFI and SD-AV microcapsules, respectively.

SEM micrographs of the OFI and AV mucilages are presented in Figure 3, while the SEM micrographs for the SD-OFI and SD-AV microcapsules are shown in Figure 4.

As shown in Figure 3a, the surface structure of the OFI mucilage, captured with a magnification of 500×, was characterized by an irregular, compact, dense, and cracked morphology. In comparison, the surface structure of the AV mucilage (Figure 3b) had a rough, scaly, and porous morphology. The images of the mucilage which were taken with a magnification of 5000×, revealed the presence of small particles, possibly corresponding to protein aggregates adhered to the carbohydrate blocks in the OFI sample (Figure 3c). The images of the AV sample (Figure 3d) show a heterogeneous and slightly rough morphology with some cavities. These types of morphologies have been associated with the conditions used during the extraction and flocculation processes of the mucilage during ethanol precipitation as well as the drying conditions of the sample [39,40].

The SEM micrograph of the surface structure of the SD-OFI microcapsule, observed with a magnification of 500× (Figure 4a), shows an agglomeration effect (adherence) between the particles. This is consistent with a phenomenon of attraction by electrostatic and van der Waals forces that is characteristic of samples with a high amount of carbohydrates [15]. A similar morphology was observed in microcapsules of betaxanthins from orange *Opuntia megacantha* fruits using a mixture of maltodextrin-cactus cladode mucilage as encapsulating agents [41]. On the other hand, the surface of the SD-AV microcapsule (Figure 4b) showed less agglomerate formation, which can be attributed to a greater resistance to the hot air flow during the drying process [41].

The SEM micrographs observed with a magnification of 5000× show particles that were spherical with a cracked and dented morphology in the SD-OFI sample. The cracking and bulging irregularities in these microcapsules can be attributed to the desorption of the air by the droplets and the shrinkage of the particles during the drying process [20]. Similar morphology has been reported for Lacto-bacillus acidophilus La-05 microcapsules protected with flaxseed mucilage and a soluble protein [42]. On the other hand, the SD-AV microcapsules showed an irregularly particle shape (spherical-type), and most of them without apparent damage. This morphology is responsible for discouraging the entry of oxygen into the capsule, thus increasing the stability of the bioactive (β-carotene) compound [43]. However, some of the particles presented a dented morphology with roughness surfaces, which was attributed to the shrinkage of the particles during the drying process typical of microcapsules produced by the spray-drying process [19].

**Figure 5:**
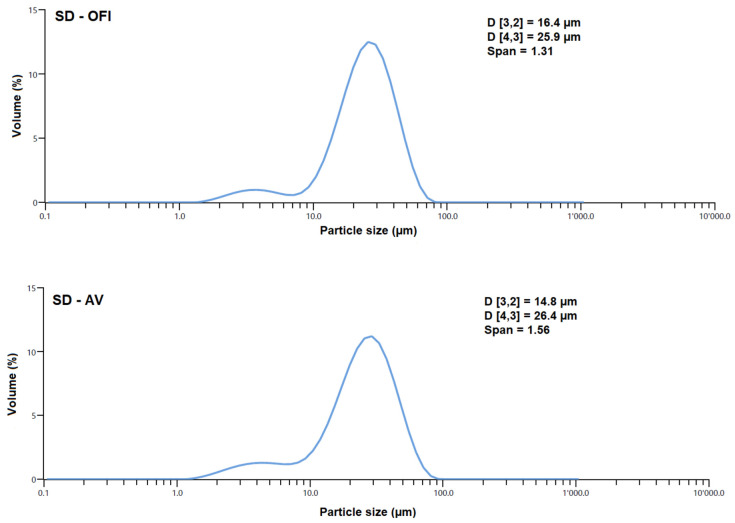
Particle size distribution plots of SD-OFI and SD-AV microcapsules. D[3,2] is area-volume mean diameter and D[4,3] is volume weighted mean diameter calculated by the software of the equipment used.

The particle size distribution and the average diameter of the SD-OFI and SD-AV microcapsules are shown in Figure 5. Both types of microcapsules showed a bimodal behavior, i.e., two maxima, with an average maximum particle size of 25.9 (SD-OFI) and 26.4 μm (SD-AV). A similar particle size, around 25.3 µm, was observed in Lactobacillus casei microcapsules using Alyssum homolocarpum mucilage and inulin as wall materials [44]. This mean particle diameter for the SD-OFI and SD-AV microcapsules was related to the size of the droplets formed from the input emulsions during the spray-drying and the viscoelastic characteristics of the mucilage wall materials [45]. The span values of the SD-OFI and SD-AV microcapsules were 1.31 and 1.56, respectively, indicating a uniform particle size distribution and greater homogeneity in both cases. These results are in agreement with those reported by Campo et al. [46], who observed an increase in span values with the addition of high amounts of chia oil in chia seed mucilages nanoparticles.

### 3.5. Thermal Properties

**Figure 6:**
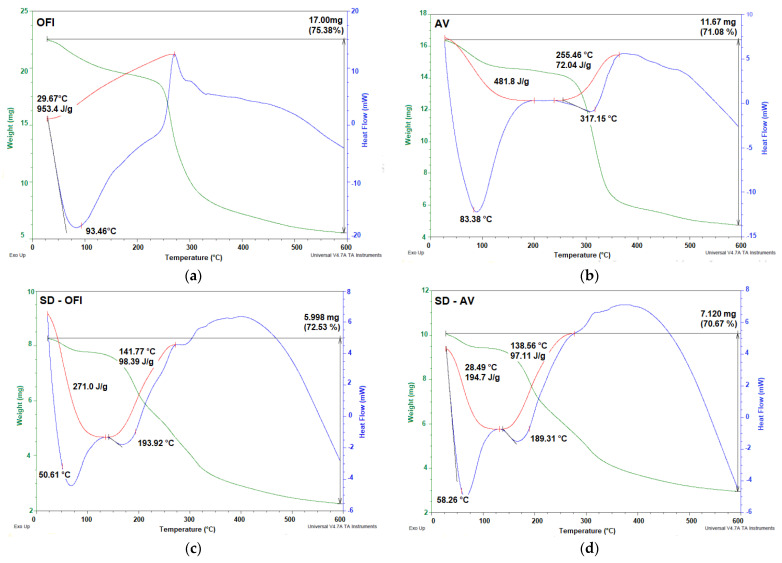
TGA/DSC thermograms of OFI (**a**) and AV (**b**) mucilages, and SD-OFI (**c**) and SD-AV (**d**) microcapsules.

The thermal behavior of the OFI and AV mucilages, as well as the SD-OFI and SD-AV microcapsules, are shown in Figure 6.

The thermograms of the OFI and AV mucilages showed the typical thermal characteristics reported for these biomaterials [16]. Both SD-OFI and SD-AV microcapsule samples showed similar thermal behavior. The thermogram of the SD-OFI and SD-AV microcapsule materials was characterized by two endothermic events. The first event occurred between 30 and 150 °C (Tg of 50.61 °C for SD-OFI and 58.26 °C for SD-AV) with a weight loss of less than 5%. This was attributed to the evaporation of the water adsorbed and structurally incorporated into theses samples. The presence of water in these samples was attributed to the hydrophilic nature of the functional groups of the polysaccharides present in the OFI and AV mucilages. The Tg values represent the interaction and the crosslinking density between the components of the guava pulp and the wall materials, as well as the stiffness, structure of the polymer chain, and molecular weight of the species contained in the microcapsules [47]. The second thermal event occurred between 150 and 200 °C (with a peak of 193.92 °C for SD-OFI and 189.31 °C for SD-AV), with a mass loss of 67.53% (SD-OFI) and 65.67% (SD-AV). This was attributed to the decomposition/volatilization of the microcapsule material. Similar thermal behavior was observed in annatto extract microcapsules using Psyllium mucilage as a wall material [48]. In general terms, our thermal analysis revealed that SD-OFI microcapsules were slightly more thermally stable than SD-AV microcapsules due to their higher melting and degradation temperature.

## 4. Conclusions

The present study revealed the possibility of microencapsulating pink guava carotenoids by spray-drying (SD) using natural hydrocolloids (mucilages) extracted from cladodes of *Opuntia ficus-indica* (OFI) and aloe vera (AV) leaves as wall materials. This microcapsule formulation was possible given the good emulsifying capacity of the OFI and AV mucilages, allowing at the same time to protect against oxidation and by-pass the hydrophobic nature of guava carotenoids. The stability of the feed spray-drying emulsion was determined by the properties of the microcapsules. Meanwhile the concentration of the encapsulating agent impacted the characteristics of the microcapsules. The SD-AV microcapsules presented a higher content of 11.2 µm/g-dry-sample of carotenoids and 14% more antioxidant capacity with an irregular (almost spherical) particle morphology without apparent damage. In contrast, the SD-OFI microcapsules showed a greater thermal stability and dietary fiber content (29% higher). Red guava pulp microcapsules using OFI and AV mucilages as encapsulating material can be a valued source of bio-functional natural ingredients (colorant with anti-radical power) to be incorporated into food products with benefits for the health of the consumer. These microcapsules may have important commercial applications in the future in the health-promoting natural additive market. For instance, they can be applied as food fortification ingredients, due to the wall material rich in dietary fiber that can provide therapeutic effects different from those provided by carotenoids (i.e., an antioxidant capacity). An important direction of this research was the acquisition of polymers from the recovery of food by-products, a current trend in microencapsulation by spray-drying.

## Figures and Tables

**Table 1:** Physicochemical characterization of lyophilized guava pulp (LGP) and guava pulp microcapsules (SD-AV and SD-OFI). Different letters in the same row and column for each parameter indicate a statistical difference (*p* < 0.05) between samples.

Parameter	LGP	SD-AV	SD-OFI
TCC ^1^	190.9 ± 0.2 ^a^	42.6 ± 0.2 ^b^	31.4 ± 0.3 ^c^
TEAC ^2^	32.2 ± 0.3 ^a^	26.8 ± 0.2 ^b^	23.2 ± 0.2 ^c^
Total dietary fiber ^3^	-	22.8 ± 0.1 ^b^	32.1 ± 0.1 ^a^
Color parameters			
L* (luminosity)	70.83 ± 0.02 ^b^	68.94 ± 0.01 ^b^	77.80 ± 0.02 ^a^
a*	13.07 ± 0.04 ^a^	7.48 ± 0.02 ^b^	5.89 ± 0.02 ^c^
b*	24.27 ± 0.01 ^b^	31.61 ± 0.03 ^a^	22.39 ± 0.01 ^b^
C_ab_* (chroma)	27.29 ± 0.02 ^b^	31.78 ± 0.03 ^a^	22.56 ± 0.02 ^b^
h_ab_* (hue)	61.55 ± 0.03 ^b^	77.27 ± 0.01 ^a^	77.19 ± 0.01 ^a^

^1^ TCC is represented as µg β-carotene/g of sample in dry base. ^2^ TEAC is represented as mmol Trolox equivalents/kg of sample in dry base. ^3^ Expressed as g/100 g.

## Data Availability

The data presented in this study are available on request from the corresponding author.
